# Oropouche Fever: A Review

**DOI:** 10.3390/v10040175

**Published:** 2018-04-04

**Authors:** Hercules Sakkas, Petros Bozidis, Ashley Franks, Chrissanthy Papadopoulou

**Affiliations:** 1 Department of Microbiology, Faculty of Medicine, School of Health Sciences, University of Ioannina, 45110 Ioannina, Greece; pbozidis@cc.uoi.gr (P.B.); cpapadop@cc.uoi.gr (C.P.); 2 Department of Physiology, Anatomy and Microbiology, La Trobe University, Bundoora, VIC 3086, Australia; A.Franks@latrobe.edu.au

**Keywords:** Oropouche virus, OROV disease, arbovirus, Culicoides, Amazon

## Abstract

Oropouche fever is an emerging zoonotic disease caused by Oropouche virus (OROV), an arthropod transmitted Orthobunyavirus circulating in South and Central America. During the last 60 years, more than 30 epidemics and over half a million clinical cases attributed to OROV infection have been reported in Brazil, Peru, Panama, Trinidad and Tobago. OROV fever is considered the second most frequent arboviral febrile disease in Brazil after dengue fever. OROV is transmitted through both urban and sylvatic transmission cycles, with the primary vector in the urban cycle being the anthropophilic biting midge *Culicoides paraensis*. Currently, there is no evidence of direct human-to-human OROV transmission. OROV fever is usually either undiagnosed due to its mild, self-limited manifestations or misdiagnosed because its clinical characteristics are similar to dengue, chikungunya, Zika and yellow fever, including malaria as well. At present, there is no specific antiviral treatment, and in the absence of a vaccine for effective prophylaxis of human populations in endemic areas, the disease prevention relies solely on vector control strategies and personal protection measures. OROV fever is considered to have the potential to spread across the American continent and under favorable climatic conditions may expand its geographic distribution to other continents. In view of OROV’s emergence, increased interest for formerly neglected tropical diseases and within the One Health concept, the existing knowledge and gaps of knowledge on OROV fever are reviewed.

## 1. Introduction

Oropouche fever is an emerging zoonotic disease caused by Oropouche virus (OROV), an arbovirus of the Orthobunyavirus genus in the *Peribunyaviridae* family (order Bunyavirales), which is transmitted to humans predominantly by the biting midge *Culicoides paraensis* [1,2]. OROV fever is an acute febrile disease, similar to dengue fever, with common clinical symptoms such as fever, headache, muscle and joint pain and skin rash, which may develop into meningitis and/or encephalitis [3,4]. OROV was first isolated in 1955 from the blood of a forest worker in Vega de Oropouche, Trinidad and from then onwards it has been found circulating in the Amazonian region (South and Central America) causing sporadic cases and outbreaks. Since the early 1960s, OROV has been implicated in more than 30 epidemics. However, it is only recently that OROV fever has attracted more research attention for reasons associated with the climate change, the geographic expansion of the arthropod vectors, the globalization of human and animal transportation and the emergence of other arboviral diseases hitting the headlines (West Nile fever, Zika). Furthermore, OROV’s potential to spread geographically, increasing the probabilities of the disease to emerge in new areas is signifying its importance at an international public health level. Because of its potential public health significance, an update of the existing knowledge on OROV fever is presented.

## 2. The Virus

OROV is a member of the Orthobunyavirus genus that comprises more than 170 viruses of 19 different serogroups and 48 species complexes [4,5], including viruses with great human and veterinary importance, such as La Crosse, Akabane, Cache Valley and Schmallenberg [6]. OROV belongs to Simbu serogroup, which consists of 25 viruses classified into seven complexes and two phylogenetic subclades, named Manzanilla and Oropouche (subclade A), Simbu, Akabane, Sathuperi, Shamonda, and Shuni (subclade B) [5].

One of the important characteristics of the Simbu serogroup viruses is their high genetic diversity, which is attributed to their wide geographical distribution. According to recent studies, this trait is most likely associated with divergences in either the vectors or the definitive hosts, or in both, underpinned by variations in the M segment of these viruses due to re-assortment or adaptation to diverse environments [4,6,7]. Of the subclade A viruses, 77% have been isolated from the Americas, with species also isolated from Australia, South Africa and Vietnam [7]. The Oropouche complex is the only one that includes species affecting humans namely the OROV, Jatobal, Iquitos, Leanyer, Oya and Thimiri viruses [4,7].

**Figure 1:**
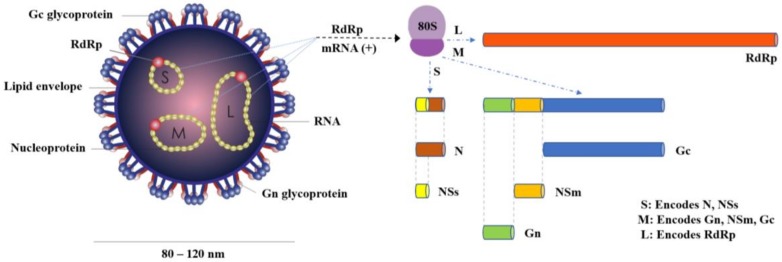
Schematic diagram of OROV particle and genome structure.

OROV is a negative-sense, single-stranded RNA virus with a spherical lipid-enveloped genome, sized from 80 to 120 nm in diameter, consisting of three single stranded RNA segments called large (L), medium (M) and small (S), surrounded by helicoidal nucleocapsid and encoding the viral RNA-dependent RNA polymerase (RdRp), the viral surface glycoproteins (Gn and Gc) and the nucleocapsid protein (N), respectively [8]. Two more proteins, the nonstructural NSm and NSs, are also encoded by the OROV genome, the first by the M segment and the latter by the S segment [8] (Figure 1).

Through phylogenetic analyses, three virus genotypes (I, II, III) were identified in 2000, from Brazil, Trinidad, Peru and Panama, respectively [9], and, ten years later, a fourth genotype (IV) was reported in Brazil [10]. Genotype I includes Brazilian strains isolated in the states of Acre, Amazonas, Maranhȃo, Tocantins, Pará, Trinidad and Tobago and is the most widespread in Brazil [11,12]. Genotype II includes strains isolated in the states of Amapá, Pará, and Rondônia in Brazil, as well as strains from Peru. Genotype III includes strains isolated in Acre, Minas, Gerais and Rondônia in Brazil and strains from Panama. Genotype IV includes strains isolated from Amazonas state in Brazil [10,13]. According to the study performed by Vasconcelos et al. (2011), Genotype I includes three sub-genotypes (Ia, Ib, Ic), Genotype II another 3 (IIa, IIb, IIc), Genotype III includes 2 (ΙIIa and IIIb), while the genetic divergence (nucleotide sequence) within the first three Genotypes ranges from 3% between I and II to 4.4% between I and III, and, for the fourth Genotype, it ranges from 5.3% between IV and I to 6.8% between IV and III, with the mean genetic divergence among the four OROV lineages to be 4.6% [10]. The existence of four OROV lineages, genotypes and sub-genotypes, has significant biological dimensions on this virus evolution and dispersion route; chronologic analysis of the N gene, epidemiological data and lineage definition confirmed the monophyletic origin of the OROV, which distinguishes this virus from other members of the Simbu group and shed light into the origin and dispersal of OROV in South and Central America. The estimated emergence dates for Genotype I that were first to appear were estimated to be approximately 112 years ago, for Genotype II almost 91 years ago, for Genotype III about 37 years ago and for Genotype IV nearly 43 years ago [10].

The high levels of genetic similarity observed in members of the OROV species are associated to re-assortment events amongst these species, occurring during many occasions of host and vector range changes, and pathogenicity variations [7]. Re-assortment in multi-segmented viruses is regarded as one of the principal mechanisms initiating creation of an offspring virus containing an assortment of L, M and S segments. Such re-assortment events arise when two genetically related viruses are infecting simultaneously one single cell [5]. An analogous re-assortment event between Madre de Dios virus and OROV might explain their isolation from the same geographical region. Recently, re-assortments involving viruses of the Manzanilla species and members of the Oropouche species such as Jatobal and Iquitos viruses with OROV have been described [7,14]. The genetic exchange between a highly infectious and a highly pathogenic virus has the potential to result in a reassortant with very high infectivity and pathogenicity, but this assumption cannot be documented with currently available data [15]. Nevertheless, monitoring for the prevalence of viral re-assortment events is important, taking into account potential evolutionary implications, particularly for viruses infecting mammals.

## 3. Arthropod Vectors

OROV’s predominant vector *C. paraensis* belongs to genus *Culicoides* (Order: Diptera, Family: Ceratopogonidae), which includes over 1400 species distributed worldwide, except the polar regions and New Zealand [16]. *Culicoides* includes 32 subgenera and 38 groups, yet 13% of the species remain unclassified [17]. The *Culicoides* genus is of major international public health interest because it includes species that are vectors of arboviruses of human and veterinary importance; the majority (96%) of these species are hematophagous, feeding on humans and other mammals such as sheep, goats, cattle, horses, deer, and birds [16,18]. More than 50 different viruses have been isolated from *Culicoides* midges worldwide, many of them of major veterinary interest such as the Bluetongue virus (BTV), Equine encephalitis virus (EEV), Epizootic hemorrhagic disease virus (EHDV), Akabane virus (AKAV), Bovine ephemeral fever virus (BEFV), African horse-sickness virus (AHSV), Palyam viruses and Schmallenberg virus [16,18,19,20,21]. The 2011 emergence of Schmallenberg in Europe and the recorded spread and persistence of BTV northwards in Europe, in areas where the two viruses had never been reported, demonstrate the ability of the vector to expand its geographic distribution.

The actual host range of *Culicoides* midges remains to be determined, but existing data indicate that they are mainly mammalophilic and/or ornithophilic, blood-feeding on various mammals and birds, depending on the availability of these hosts [20]. The host preference and feeding behavior of *Culicoides* midges have been investigated using either laboratory methods such as serological analysis of abdominal blood by precipitin test, enzyme linked serological assays (ELISA) and polymerase chain reaction (PCR) or observational studies based on collection of adult female midges by light, sticky or animal-baited traps or by direct aspiration from animals, the latter being the most credible study method [22]. Quantitative data linking the feeding rates to host availability are scarce, while it is quite clear that many of the *Culicoides* species can be opportunistic in host preference [18]. Only female adult midges take blood meals, which are necessary for the maturation of their fertilized eggs, males never feed on blood [16,23]. Eggs are laid in moist places and hatch into larval stage in 3–10 days, but much of the life-cycle of *Culicoides* related to larval stages remains unexplored. *Culicoides* breed in habitats that provide the proper moist environment for the eggs to survive and hatch, like mud at the soil-water interface, dung pats, highly organic and moist soil substrates such as manure mixed with soil, which can accommodate abundant vectors very close to livestock, thus facilitating arbovirus transmission to animals and to humans living in the proximity of animal farms [18].

*C. paraensis* is reported to be abundant during the hot or rainy months of the year and is characterized by broad geographic distribution, a feature attributed to its ability to survive and reproduce in semi-urban regions that are close to areas of high human density [24,25,26,27]. The larvae of *C. paraensis* develop in various habitats capable of remaining moist enhancing larvae feeding in dry periods, like rainforests, riverbanks, damp soil, tree holes, decaying organic matter of plant origin such as rotting banana and plantain stalks, stumps and cacao husks [27,28,29,30]. *C. paraensis* prefer biting indoors during day and night, more often by late afternoon, with a reported indoor/outdoor ratio of 29% [31], during light to torrential rains [30]. In addition, *C. paraensis* bites have been reported to be implicated in common allergic reactions and skin irritation, like dermatitis, eczema or scars [19].

OROV is also distributed to areas where hematophagous mosquito species *Culex quinquefasciatus*, *Coquillettidia venezuelensis*, and *Aedes (Ochlerotatus) serratus* can breed and may be naturally infected by the virus [1,32,33]. It is well documented that, in 1960, OROV was isolated from mosquito species *Cq. venezuelensis*, and *Ae. serratus* in the Trinidad and Amazonian region of Brazil, respectively [5]. Both species are considered as secondary vectors of the virus and they are found at high densities in sylvatic environments [34,35]. *Cq. venezuelensis* is reported to be breeding in aquatic ecosystems, such as pools, lakes and rivers, characterized by nocturnal biting activity [35], while *Ae. serratus* demonstrates diurnal biting activity, feeding preferably from large mammals [34]. *Cx. quinquefasciatus* (member of *Cx. pipiens complex*) commonly known as “southern house mosquito”, is one of the most widespread mosquitoes in tropical and subtropical areas, feeding in both animals and humans, occurring most frequently in urban environments [36]. It is considered a less important anthropophilic vector for OROV [4,36], and its competence and distribution has been related to variable climatic conditions, such as rainy seasons and low temperatures. It usually breeds in organically rich and polluted surface waters or artificial containers, its activity usually begins at night, biting humans, amphibians, pigs, birds, horses, cattle, sheep, dogs and rabbits [37].

## 4. Transmission Cycles

**Figure 2:**
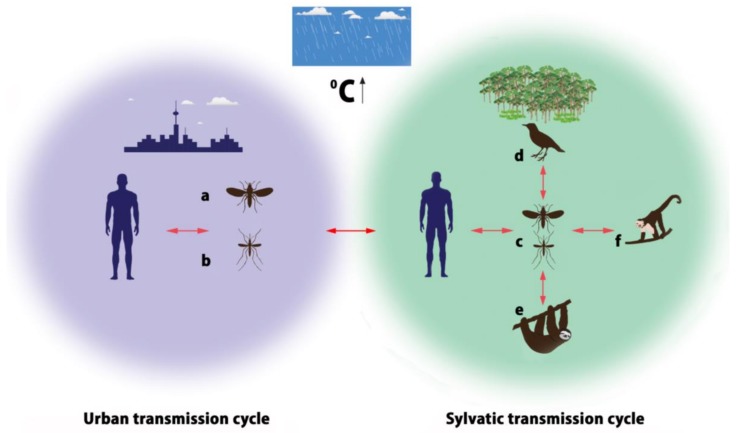
Urban (U) and sylvatic (S) transmission cycles of OROV (Vectors—a: *C. paraensis*; b: *Cx. quinquefasciatus*; c: *Culicoides* midges, *Cq. venezuelensis, Ae. serratus, Cx. quinquefasciatus*; Hosts—d: birds; e: sloths; f: monkeys).

OROV is conserved in nature by an urban cycle and a sylvatic cycle, which may include several different vectors (Figure 2). In the urban cycle, *C. paraensis* is the primary vector [38], which has been implicated in large epidemics affecting up to 100,000 patients [39]. The role of *C. paraensis* as a main urban vector has been supported by experimental and epidemiological data. Pinheiro and colleagues proved that *C. paraensis* midges are able to transmit OROV to hamsters 6 to 12 days after blood-feeding on viremic patients, and the threshold titer enabling transmission and infection was approximately 5.3 log_10_ SMLD_50_/mL [32]. It has been reported that other *Culicidae* species, such as *Cx. quinquefasciatus* can contribute to the OROV transmission, but relevant experimental studies show the threshold of infection for this species to be high (≥9.5 log_10_ SMLD_50_/mL) indicating low efficiency of virus transmission by this mosquito species [40]. The *C. paraensis* efficiency to transmit the disease feeding on lower virus-titer blood-meals provides strong evidence that they are the most important OROV vectors [24]. Although *Cx. quinquefasciatus* is recognized to be an anthropophilic vector to a lesser extent, OROV is the only member of the *Orthobunyavirus* genus isolated from this mosquito species, based on the detection of OROV’s SRNA in both patients and insects by nested-reverse transcription-polymerase chain reaction (RT-PCR) [4]. The epidemiological data are based on observations that higher incidence of human OROV infections is recorded in areas with high densities of midges [40] and during epidemics midges’ densities are higher too [5]. Relevant studies have excluded involvement of domestic animals such as cats, dogs or chickens, in the urban cycle, suggesting humans to be the sole vertebrate host, with no evidence of direct human-to-human OROV transmission [5]. Hemagglutinin antigens to OROV obtained from hamster brain and serum samples have been used as serological diagnostic tools for epidemiological surveys as well [5].

In the sylvatic cycle mammals, wild and domestic birds are the natural reservoir hosts. Antibodies to OROV have been found in pale-throated three-toed sloths (*Bradypus tridactylus*), nonhuman primates such as capuchin monkeys (*Sarajus* spp.), black-and-gold howler monkeys (*Alouatta caraya*), black-tufted marmosets (*Callithrix penicillata*), rodents (*Proechimys* spp.), and birds (*Fringillidae*, *Thaurapidae*, *Columbidae*) implicated in the transmission of OROV. Nevertheless, the existing literature on OROV detection in the reservoir hosts is rather limited. Published data report OROV isolation from one sloth in 1960 (Brazil), antibodies detection in one rodent, 34 wild birds and 12 domestic birds during an epidemic in Mojui dos Campos, Pará State, Brazil in 1975 [40,41], isolation from non-human primates in Arinos region, Southeast Brazil in 2000 [11] and in Miranda, State of Mato Grosso, Brazil in 2013 [42]. In a very recent publication by Romero-Alvarez and Escobar (2017), only sixteen references related to OROV detection in different reservoirs in Brazil, Colombia, Trinidad and Venezuela are cited [43]. The mosquito species *Cq. venezuelensis*, *Ae. serratus*, *Cx. quinquefasciatus* and midges of the genus *Culicoides* have been reported to be likely vectors in the sylvatic cycle as indicated by epidemiological and laboratory based evidence [4,33,41,43,44].

Humans are probably the link between the two distinct transmission cycles, since OROV is usually invading urban areas through a viremic individual who visited a forest, got infected there and returned to an urban area during the viremic phase [5]. Still, there are gaps of knowledge in the transmission cycle, such as the reported low isolation rates (1:12,500) of OROV from midges during epidemics, which may be attributed to low susceptibility of this vector to the virus or to the ability of a fragment only of the insect population to transmit OROV, but this has to be answered by further studies [5,40]. In addition, the role of wild animals has not been explored adequately in other geographic locations outside Brazil, as well as the role of birds in the potential spread of OROV in other countries or continents taking into account the birds’ wide geographic distribution and movement. From the available literature data, OROV is circulating at low levels in the wildlife and human reservoirs, but whenever a perturbation in the environment (vegetation loss/deforestation, habitat loss) and/or in the community (human and/or animal immigration) occurs, OROV outbreaks are emerging. Therefore, the urban transmission of OROV can probably be sporadic and temporary, but also may be maintained among urban populations for longer periods or permanently, at a relatively low level of activity and thus may go unrecognized [29]. A surveillance study in Iquitos, Peru, during a period of significantly increased transmission, suggested a peri-urban transmission cycle, since the majority of OROV isolates were detected in both rural and urban sites located in the outskirts of the city [45].

## 5. Disease Epidemiology

**Figure 3:**
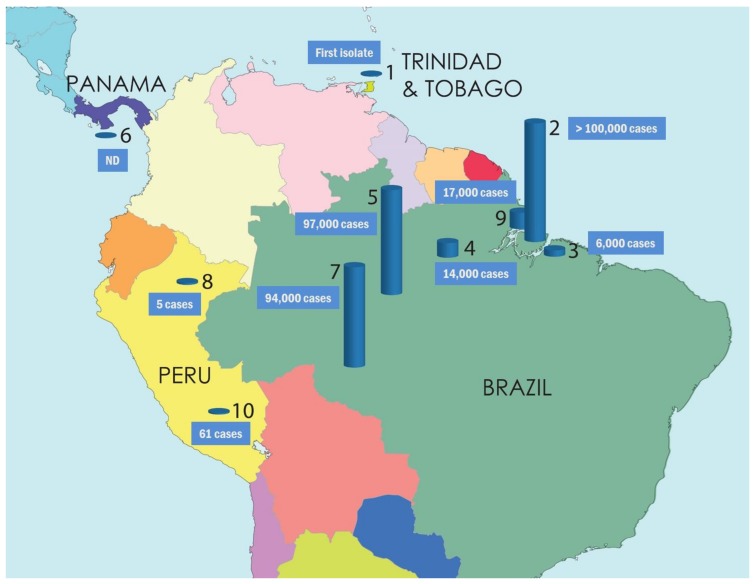
The spread of OROV in Central and South America from 1955 to 2016 (Bar symbols represent the affected populations in different geographical locations, and the estimated or confirmed number of infected individuals in several outbreaks). 1—Trinidad and Tobago, 1955 first isolated (one case); 2—Belém, Pará, Brazil, 1961 first epidemic (11,000 cases), 1979–1980 epidemic (>100,000 cases); 3—Braganca, Pará, Brazil, 1967 epidemic (6000 cases); 4—Santarém, Pará, Brazil, 1975 epidemic (14,000 cases); 5—Manaus, Amazonas, Brazil, 1980–1981 epidemic (97,000 cases); 6—Panama, 1989 first epidemic (N.D: no data); 7—Ariquemes and Ouro Preto do Oeste, Rondônia, Brazil, 1991 epidemic (94,000 cases); 8—Iquitos, Peru, 1992 first epidemic (five confirmed cases); 9—Magalhães Barata & Maracanã, Pará, Brazil, 2006 epidemic (17,000 cases); 10—Cusco, Peru, 2016 last reported epidemic (61 cases).

The emergence and re-emergence of OROV fever in Central and South America have resulted in more than 30 epidemics in Brazil, Peru, Panama, and Trinidad and Tobago, the majority being reported in Brazil, with disease prevalence to be 20% in both urban and rural human populations of the affected regions (Figure 3) [46,47,48]. OROV is the second most frequent arbovirus in Brazil after dengue virus and among 200 different arboviruses isolated in this country [49,50], with considerable social and economic impact. It is estimated that over half a million of people have been infected by OROV in over 60 years, but it is likely the actual number of cases to be higher since many of them may go undiagnosed or misdiagnosed due to similar clinical manifestations with other febrile diseases caused by other arboviruses (e.g., dengue, West Nile, yellow fever, Zika, chikungunya, Guama, Mayaro) co-circulating in the endemic regions [5,48,51,52].

As the recorded number of OROV outbreaks and sporadic cases are increasing in Brazil, it is speculated that it will continue to occur in the Amazonian region over time [39] for reasons including climate and environmental changes, animal and human dislocations, more efficient surveillance and diagnostic tools, and increased research interest. Furthermore, Brazil is a tropical country, the fifth most populated in the world, with inhabitants living mostly in large densely populated cities infested by various species of mosquitoes. OROV-infected humans and animals moving into big cities from endemic regions are increasing the risk for more and larger outbreaks. A great part of Brazil’s territory is covered by rainforests and ecosystems, containing an exceptionally diverse fauna and flora that can maintain zoonotic arboviruses. Particularly, the Brazilian Amazon region is providing perfect conditions for OROV’s maintenance. The tropical climate is ideal for propagation and conservation of the arthropod vectors, and anthropogenic land changes (deforestation, changes in land use/plantations, building of motor highways and dams) have a tremendous impact on the habitats of the reservoir hosts, forcing the latter to move closer to urban and peri-urban regions where the vectors are proliferating. The environmental changes are driving people to dislocate and move towards urban or neo-urbanized regions offering better working and living opportunities, often settling close to new plantation farms (banana or other exotic fruit monoculture plantations) created after deforestation, where the virus’s reservoir hosts either pre-existed or have settled there as well [5,10,11,28,29,43]. Considering that environmental and climate changes, and extensive animal and human population migration are a global phenomenon, it will not be surprising for OROV to spread outside South America in the near future.

The epidemiological history of OROV fever demonstrates the role of human-induced environmental and climatic changes on hosts’ and vectors’ spread and disease prevalence. For the first time, OROV was isolated in 1955 from a febrile human case in the island of Trinidad in the Caribbean Sea; the infected patient was a forest worker and during that incident neutralizing antibodies were detected in the blood of a few more forest workers and from a number of cebus and howler monkeys that were living in Trinidad [1]. Five years later (1960), OROV was isolated from a pool of *Cq. venezuelensis* mosquitoes in Trinidad; interestingly, in the same year, the virus was isolated from a sloth (*Bradypus tridactylus*) and from *Ae. serratus* mosquitoes in Brazil, both hosts being captured near a forested area during the construction of the Belém-Brasilia highway [5,10]. Within a year (1961), the first recorded OROV fever outbreak (~11,000 cases) occurred in Belém, Pará State, the most populous state of Northern Brazil, well known for its rainforests and the Amazon river [4,32,39]. Infection was identified by both neutralization and complement fixation tests [32]. Between 1961 and 1978, seven outbreaks of OROV fever were reported in small and large urban centers in Pará State and around 30,000 persons were infected [32]. During the outbreak in Santarém (1975), serum samples were tested against the BeAn 19991 strain by hemagglutination inhibition (HI) assay, which was routinely used in most of these epidemics [38,41]. It is reported that, during those outbreaks, in the largest urban centers, OROV was limited to certain districts, but, in villages, it was spread throughout, while *C. paraensis* and *Cx. quinquefasciatus* were the most common vectors in the endemic areas [32,41]. In 1980–1981, the two largest epidemics of OROV fever (>100,000 people infected) were recorded in Belém (Pará State) and in Manaus (Amazonas State) [29], which is located in the middle of the Amazon rainforest, and, like Belém, is densely populated. During the 1980 outbreak in Pará State, neurological symptoms defined as meningitis or meningismus were manifested in 4.1% of the cases associated with OROV infection [53].

From 1980 to 2005, only sporadic cases or self-limited outbreaks were reported in the Brazilian Amazon regions, mainly in small villages indicating potential silent circulation of the virus [54]. In most of the cases, laboratory diagnosis was achieved serologically by an HI test and IgM antibody capture enzyme-linked immunosorbent assay (MAC Elisa) [54,55,56]. During 1980–2004, OROV fever spread to other northern (Amapá, Acre, Rondônia, Tocantins) and northeastern (Maranhão) Brazilian states [11,54]. In 2000, the first isolation of OROV from the monkey species *Callithrix* sp., in southeastern Brazil (outside the known epidemiological area of virus transmission), demonstrated the OROV’s potential to spread to new hosts and new areas [11]. OROVs expand in southeastern regions includes the risk of larger epidemics because they are the most densely populated part of Brazil. Rio de Janeiro, São Paulo, and Belo Horizonte are located there, which are distinguished by intense migration, improved transportation fostering zoonoses dissemination and massive tourism attracting visitors from all over the world, who may be infected and spread OROV outside Latin America.

In 2003–2004, two small outbreaks of OROV fever occurred in urban and rural municipalities of Pará State (Parauapebas, Porto de Moz). In 2006, a large epidemic (18,000 individuals infected) was recorded in two more municipalities (Magalhães Barata, Maracanã) signifying re-emergence of OROV in Pará state after 26 years. The observed epidemic gap was attributed to possible accumulation of OROV susceptible people not previously exposed to the virus (immigrants, younger populations). Serological surveys performed among febrile patients in Parauapebas and Porto de Moz revealed that 20.7% and 46.7%, respectively, had HI antibodies and 22.3% and 52.1%, respectively, had IgM antibodies to OROV [13]. A similar surveillance study in Magalhães Barata and in Maracanã indicated that 76.7% of the febrile patients had specific HI antibodies and 73.3% had IgM-ELISA antibodies [54]. OROV genotype II was responsible for the epidemic incidents in all municipalities where the virus was isolated from patients [13,54]. Taking into consideration that genotype II had been isolated in 1991 in Rondônia (western Amazonian region) and during the 1990s in Peru, it appears that this genotype is transitioning from western to eastern Amazonian regions.

In 2007–2008, OROV outbreaks were recorded in Manaus and infection was detected by an in-house enzyme immunoassay for IgM antibodies in acute-phase serum samples, using infected cell culture as antigen (EIA-ICC) [3]. In 2011–2012, new outbreaks were reported in several municipalities of Mato Grosso State (Western Brazil) and the laboratory diagnosis was accomplished by nested RT-PCR [4]. Mato Grosso is surrounded by states where OROV is endemic, has a high rate of population growth due to timber, ranching and agricultural development, and is acclaimed for its intriguing biodiversity and the world’s largest wetlands; in fact, the state has all the distinctive features favoring vectors’ preservation and OROV maintenance [57]. In 2016, the first OROV fever case outside the Amazon region was reported [33]. The OROV antigen was detected in peripheral blood leukocytes by indirect immunofluorescence from two febrile patients in São Paolo, raising concerns on the occurrence of OROV fever out of the Amazon region [33]. The broad distribution of the primary vector *C. paraensis* from South and Central America to northern United States of America, coupled with anthropogenic environmental disturbances and extensive human and animal travelling accentuate the potential for OROV to emerge in other territories. From the reviewed literature on OROV fever emergence in Brazil, it is clear that the common features of the affected areas were the tropical climate, high frequency of rains, similar activities of the residents (cattle breeding, plant monocultures), unplanned urbanization and very poor standards of living [26,28,30,57].

The first isolation of OROV virus in Panama was recorded in 1989 in the village of Bejuco from febrile patients suspected of dengue fever, during a dengue surveillance program in the greater metropolitan area of the Panama City. At that time, OROV was not known to exist in Panama, but a later retrospective serological study of sera collected in 1968 and 1978 from residents of the affected community revealed that 25% of the samples had OROV antibodies, suggesting earlier human exposure to virus and possibly infection, which had gone unrecognized [5,29,43].

In 1992, a couple of years after OROV fever was detected in Panama, the disease was detected in Peru, involving five febrile patients living in Iquitos port on Amazon River [58], and since then outbreaks have been reported in various regions of the country [30,43]. Epidemiological studies based on ELISA and plaque-reduction neutralization test (PRNT) have demonstrated high seroprevalence and continuous transmission amongst the population of Iquitos [59,60], which is the largest city in the Peruvian Amazon, serving as a touristic, trade and military center of the greater region, with intense human and animal population transition favoring OROV fever transmission [30]. The recorded sporadic cases and self-limited outbreaks reported in the Peruvian Amazon regions during 1980–2005 indicate subclinical and sporadic circulation of the virus [12,54]. The most recent Peruvian outbreak of OROV fever occurred in 2016 in Cusco in southeastern Peru [43]. Epidemiological studies based on serology were conducted in the Peruvian Amazon region for determination of OROV antibody prevalence, incidence of infection and risk factors. The seroprevalence rate was 35% for urban population, 18% for rural population and 24–46% for forest communities, while the antibody prevalence increased with age, indicating prior infection among residents and continuous transmission in the population. It is likely that OROV entered into Peru spreading across the riverbanks of Amazon River assisted by intense human and animal mobilization [59].

Recent studies based on epidemiological surveillance in humans and wild mammals have demonstrated circulation of OROV in Argentina, Bolivia, Colombia, Ecuador and Venezuela [45,61,62,63].

## 6. Pathogenesis

It is recognized that OROV is highly detectable in blood from the onset of infection, and can progressively reach the neural routes, resulting in a systemic infection and primary central nervous system (CNS) inflammatory response [5]. Since the disease is systemic, and, in severe cases, the CNS is affected, with the virus being detected in cerebrospinal fluid (CSF), the relative research is mostly oriented in exploring the mechanism by which OROV is infecting CNS. Intracerebral inoculation of OROV in experimental animals, especially mice and hamsters is reported to induce the infection [64,65]. An early study on hamsters inoculated intracerebrally reports fatal severe hepatitis with necrosis of hepatocytes and Kupffer cell hyperplasia, with no detection of OROV in the animal tissues and no identification of any sites of virus replication [66]. Recent studies use the subcutaneous route for OROV inoculation into the experimental animal models because this route resembles the natural route of infection by this arbovirus. The golden hamster (*Mesocricetus auratus*), in which both the virus replication and the disease may occur, has been used for subcutaneous experimental inoculation of OROV by Rodrigues et al. (2011) [64]. Hamsters inoculated with OROV developed systemic infection with neurological motor impairment and paralysis, along with the accumulation of the virus in both brain and liver, suggesting hematogenous transmission to brain and liver. Apparently, the blood–brain barrier (BBB) is probably penetrated by a Trojan-horse mechanism considered to play significant role in several viral pathogenesis manifestations [64,67]. In this mechanism, the pathogen is carried through the blood stream hiding inside the infected phagocytes and thus it is going unrecognized to the target organs/tissues, where it can replicate, eluding any immune response, bypassing and crossing any barriers such as the BBB. However, a neural route of brain invasion may be involved due to the observed viral accumulation within neurons [64]. The hepatotropic nature of OROV is described by both Araujo et al. (1978) and Rodrigues et al. (2011), despite the different inoculation routes [64,66]. It has to be noticed that there are no reports on hepatitis manifestations in OROV fever patients, but altered liver enzymes have been reported [32].

Experimental infections in mice demonstrated severe manifestations of encephalitis related to extended spread of OROV through the brain parenchyma. In one study, suckling BALB/c mice were subcutaneously inoculated with OROV developing severe disease five days post inoculation with lethargy and paralysis leading to death >80% of the animals within 10 days. Viral replication in brain neurons pointing out the neurotropism of OROV, was documented by in situ hybridization, immunohistochemistry and virus titration [65]. Despite the severe CNS disease, histopathology was mild in brain and spinal cord with little inflammation indicating that replication in neurons may occur with relatively little functional impairment. Interestingly, spleen hyperplasia with absence of OROV recovery or antigen detection in the spleen was reported too, a finding reminiscent of the results of Araujo et al. (1978) although different animals and routes of inoculation were used, and different organs (liver, spleen) were studied [66].

Activation of mitochondrial antiviral-signaling protein (MAVS), interferon-regulatory factors (IRF) 3 and 7, and production of type I interferon (IFN-I) have been found to play an important role in the innate immune system’s response to viral infection, by controlling the virus replication, the liver damage and the progressive death in mice experimental models [46]. In addition, the interferon-regulatory factor 5 (IRF-5) has been discovered to play a key role in modulating the host antiviral response in peripheral organs that control OROV’s dissemination in CNS, having an inhibitory effect on the neuroinvasive disease manifestation and the virus replication in liver, spleen and blood during early stages of infection in mice [68]. In a later experimental study by Santos et al. (2014) in mice too, it is suggested that OROV uses the neural route during the early phases and with the progression of the infection somehow becomes able to cross the BBB, which may happen in parallel with the neural spread and probably is associated with viremia [69]. A study on OROV induced apoptosis in HeLa cells has indicated that in vitro OROV infection causes apoptosis by an intracellular pathway involving mitochondria and is triggered by a mechanism dependent on virus replication and protein expression [70]. Only recently, a minigenome and virus particle production assay was established according to BeAn 19991 and TRVL-9760 strain sequences [51]. These assays along with an OROV rescue system based on a T7 RNA polymerase-driven plasmid system developed by the same group will enhance the efforts of understanding the molecular biology of the virus [50]. Conclusively, the existing knowledge on OROV fever pathogenesis mechanism is limited and there is a need for further research in this field.

## 7. Clinical Manifestations

OROV fever is manifesting as a self-limited, dengue-like, acute febrile illness that lasts 2–7 days, and is associated with a variety of symptoms such as fever, chills, headache, myalgia, arthralgia, malaise, dizziness, nausea, vomiting, photophobia, retro-ocular pain, and, in rare occasions, skin rash appearing more commonly on trunk and arms, hemorrhagic signs such as spontaneous bleeding, petechiae, epistaxis, gingival bleeding, and CNS signs like aseptic meningitis or meningoencephalitis [5,32,39,41,43,44,47,50,53]. In some patients, physical weakness and strength loss (asthenia) have been noticed for a period of 2–4 weeks [32]. The CNS manifestations usually occur in immunocompromised individuals and children, and patients with previous BBB disruption [71]. Neurological manifestations clinically defined as meningitis or meningismus are recorded mostly during large outbreaks and include severe headache, dizziness, lethargy, diplopia, nystagmus and, in some cases, ataxia, nuchal rigidy and increased cells in CSF [44,53].

The most frequently reported symptoms as recorded during large epidemics in Brazil were: fever (100%), headache (79.3%), arthralgia (68.7%), myalgia (30%) in Parauapebas and Porto de Moz, (2003–2004) outbreak [13], fever (100%), headache (99.3%), chills (59.3%), myalgia (46.9%), dizziness (39.8%), photophobia (38.1%), and nausea/vomiting (36.3%) in the Magalhães Barata and Maracanã outbreaks, (2006) [54], fever (100%), headache (72.7%), myalgia (70.3%), arthralgia (57.8%), rash (42.2%), and hemorrhagic manifestations (15.5%), in the Manaus outbreak (2007) [3].

The disease incubation period is 3–8 days. After this period, the infected individuals develop symptoms, are highly viremic and can transmit OROV if they are bitten by midges. It is reported that viremia decreases significantly, resulting to a reduction in the viral titer at 72%, 44% and 23% on the 3rd, 4th and 5th day, respectively [4]. The acute phase of OROV fever commonly lasts 2–7 days [43,53]. Recurrence of symptoms has been observed is some cases, particularly within the first 10 days after the onset of symptoms [4,32]. In about 60–70% of reported cases, mild symptoms (fever, headache, chills) may recur one to several times 2–3 weeks after their initial manifestation [5,13,30]. The disease affects people of all age groups and both genders. In endemic countries, OROV must be included in the differential diagnosis of cases suspected of acute infection of the CNS. It is reported that 6.1% of CSF samples of hospitalized patients with confirmed viral CNS infection in Western Brazilian Amazon were found OROV positive by a combination of RT-PCR and nested RT-PCR [72,73]. It has to be pointed out that there is a lack of data on OROV’s potential effect in fetal development, although abortions and teratogenic effects in animals from other Simbu viruses have been reported [30].

## 8. Diagnosis

The clinical diagnosis of OROV fever is difficult because the disease is manifesting with symptoms similar to other infections that are circulating in the endemic areas, such as dengue, chikungunya, Zika, yellow fever and malaria. Routine laboratory blood tests are not particularly helpful, whereas the occasionally reported leucopenia and mildly elevated liver transaminase levels [32] are not specific for OROV infection. Similarly, in cases where neurological symptoms are present, the findings in the obtained CSF specimens such as white blood cells (WBC) counts, lymphocytic/monocytic type, normal to slightly decreased glucose and high protein are compatible with the majority of viral infections [71]. Thus, differential clinical diagnosis has to be based on specific laboratory tests confirming OROV fever infection.

Serologic procedures using different antigens have been routinely used for the detection of specific IgG and IgM antibodies. The available methods for laboratory diagnosis of OROV disease involve MAC Elisa, PRNT, complement fixation test (CFT), HI test [55,56,60,71,74] and EIA-ICC [3]. EIA-ICC has been also used for the detection of both IgM and IgG antibodies to OROV, in CSF samples [71]. Recently, an indirect immunofluorescence (IF) test on peripheral WBC for OROV antigen has been developed as a useful diagnostic tool [33].

The HI test is considered to have higher sensitivity but lower specificity when compared to other serological assays such as MAC Elisa, but specific antibodies can be detectable for long periods after infection [42]. Enzyme immunoassays (EIAs) using bacterially expressed recombinant nucleocapsid (rN) protein of OROV have demonstrated high sensitivity (95%), specificity (99.5%) and safety in the detection of OROV fever. In sero-epidemiological studies, the hamster serum antigen (HAS) and Vero cell lysate antigen (VCLA) have been used in EIAs for detection of specific IgG and IgM antibodies exhibiting high sensitivity and specificity (the former 93% and 99% and the latter 98% and 93%) [74]. OROV infection can be confirmed by mouse bioassays after the virus isolation from blood samples of febrile patients and subsequent intracerebral inoculation in suckling mice; however, this bioassay is time-consuming and is not indicated for routine diagnosis of the OROV infection [73].

Molecular techniques, such as nested RT-PCR, have been successfully applied as important diagnostic tools, for rapid and specific diagnosis of OROV infection in both serum and CSF samples [71,73]. A modified one-step RT-PCR was considered to be a more sensitive method than nested RT-PCR, mostly because of the smaller amplicon sizes produced by the first method and the use of the T4 phage single stranded binding protein GP32 in the reaction. The reported rate of the detected viral RNA in patient serum samples during the first five days of the disease was 93.3% instead of 26.6% by nested RT-PCR [75]. Recently, the Next-generation sequencing (NGS) technology has been suggested as a useful diagnostic tool in clinical isolates (acute-phase serum samples) with novel genome sequences sufficiently different from published data when the conventional amplification methods based on known sequences are proved to be ineffective [76]. Lately, an accurate and highly sensitive multiplexed one-step RT-qPCR was evaluated for OROV detection in cell supernatants and mouse tissues [77].

## 9. Treatment and Prevention Options

Currently, the available treatment for OROV infection is symptomatic, which means that the provided medication is only easing the symptoms but is not affecting the causative agent (by killing the virus or inhibiting its replication). Generally, the prognosis for OROV fever is good and no deaths have been reported ever since the first outbreak in 1960. In the existing literature, data on in vitro or in vivo experiments for potential treatment or prevention options are scanty. A study by Livonesi et al. (2006) on ribavirin, a broad-spectrum antiviral drug, reports no inhibitory effect on OROV and another study by Livonesi et al. (2007) reports some antiviral activity of IFN-α on OROV [78,79]. Concerning prevention options, so far, there is no vaccine for OROV fever prophylaxis in humans. Thus, prevention strategies are based on control or eradication measures for the arthropod vectors and personal protections measures. Vector control measures rely on reducing midge populations through the eradication of breeding sites, by applying good agricultural practices [27]. Personal protection measures rely on prevention of midge biting using mechanical barriers (mosquito nets), insect repellant devices (insect light traps) [80], repellant-treated clothing and anti-mosquito lotions, although the latter have been incriminated for allergies and/or dermatological reactions.

Chemical insecticides such as deltamethrin and *N*,*N*-Diethyl-meta-toluamide (DEET) have demonstrated very sufficient insecticide effects against *Culicoides* species. However, the ecological consequences of the insecticides usage at a large scale are of particular concern [81,82]. Alternatively, various natural compounds (pyrethrins, picaridin, azadirachtin) and plant derived essential oils (eucalyptus, levanter, geraniol, neem) have been proposed as repellents [27,82]. The complete and permanent eradication of the arthropod vectors is rather impossible, considering the abundance of *Culicoides* populations. Education of human populations in endemic regions (e.g., farmers, cattle-breeders, forest workers, housekeepers, pupils/students, tourists) about the seasons of high exposure risk, the potential implications on human health due to midge biting and personal protection options can contribute significantly to OROV fever prevention.

## 10. Conclusions

OROV fever is an emergent zoonotic disease that is currently endemic in certain regions of South and Central America. Despite the fact that it has been infecting over half of million people in South America, the disease—like most of the arboviral diseases—was a neglected disease for almost sixty years. Thus, data on the distribution, prevalence and incidence rates in human, animal and vector populations are inadequate and possibly underestimated. OROV fever is an acute febrile illness, but is self-limited and it is not a fatal disease, which may explain the lack of adequate attention by researchers, international health agencies and policy makers. OROV’s spread is related to environmental factors including climate, fauna and flora changes, as well as to socioeconomic factors such as human population demographics, agricultural practices and poor standards of living. There are contradictory reports on the possible impact of age and gender on the prevalence of the disease and there are no reports relating the disease severity with factors such as the geographic region, season, vector species or the virus lineage and/or genotype. Nevertheless, there is full agreement of the literature on the factors incriminated in OROV disease emergence, which are summarized in: deforestation, human population growth, intensive animal farming, monoculture plantations, highway and dam building in the tropics, climate change, increased human and animal mobilization, and globalization of food trade. The OROV fever emergence and distribution, like other vector-borne diseases, is a consequence of the ecologic equilibrium’s disturbance, which is observed world-wide. Considering that environmental, climate and demographic changes are a global phenomenon, it will not be surprising for OROV to spread outside Latin America in the near future. Furthermore, taking into account that this is another emerging zoonotic disease, it has to be encountered within the “One Health” approach through the collaboration of human and veterinary medicine, entomology, biology and environmental sciences.
